# Community Engaged Leadership to Advance Health Equity and Build Healthier Communities

**DOI:** 10.3390/socsci5010002

**Published:** 2015-12-24

**Authors:** Kisha Holden, Tabia Akintobi, Jammie Hopkins, Allyson Belton, Brian McGregor, Starla Blanks, Glenda Wrenn

**Affiliations:** 1 Department of Psychiatry & Behavioral Science, Morehouse School of Medicine, 720 Westview Drive, Atlanta, GA 30310, USA; 2 Satcher Health Leadership Institute, Morehouse School of Medicine, 720 Westview Drive, Atlanta, GA 30310, USA; 3 Department of Community Health and Preventive Medicine, Morehouse School of Medicine, 720 Westview Drive, Atlanta, GA 30310, USA; 4 Prevention Research Center, Morehouse School of Medicine, 720Westview Drive, Atlanta, GA 30310, USA

**Keywords:** community engagement, healthy communities, health equity, health disparities, community-based participatory research, ethical leadership

## Abstract

Health is a human right. Equity in health implies that ideally everyone should have a fair opportunity to attain their full health potential and, more pragmatically, that no one should be disadvantaged from achieving this potential. Addressing the multi-faceted health needs of ethnically and culturally diverse individuals in the United States is a complex issue that requires inventive strategies to reduce risk factors and buttress protective factors to promote greater well-being among individuals, families, and communities. With growing diversity concerning various ethnicities and nationalities; and with significant changes in the constellation of multiple of risk factors that can influence health outcomes, it is imperative that we delineate strategic efforts that encourage better access to primary care, focused community-based programs, multi-disciplinary clinical and translational research methodologies, and health policy advocacy initiatives that may improve individuals’ longevity and quality of life.

## 1. Health Disparities: A Global Challenge

A recent report of the World Health Organization entitled *U.S. Health in International Perspective: Shorter Lives, Poorer Health* documented the alarming implications of poor health status among many individuals, families, and communities [1]. This landmark report helps to delineate from a global perspective, comparisons among seventeen peer countries relative to the issue of life expectancy, selected medical conditions, and health outcomes particularly concerning infant mortality and low birth weight, injuries and homicides, disability, adolescent pregnancy and sexually transmitted infections, HIV and AIDS, drug-related deaths, obesity and diabetes, heart disease, mental health, and chronic lung disease. One notable and consistent finding suggested that individuals that are most negatively impacted, suffer the greatest, and highest at-risk for deleterious outcomes represent poor, underserved, and vulnerable communities inundated by individuals that live in poverty. These harsh realities warrant further examination and the critical need to determine the role of public health in the quest for global health equity.

Equity in health implies that ideally everyone should have a fair opportunity to attain their full health potential and, more pragmatically, that no one should be disadvantaged from achieving this potential [2,3]. In many nations, social justice, environmental, and economic issues may impact an individual’s livelihood, exposure to illness, and risk of early mortality according to a 2008 report of the World Health Organization’s Commission on Social Determinants of Health (CSDH) [4]. When extreme differences in health are significantly associated with social disadvantages, the differences can be labeled as health inequities; and in most cases these differences are: (1) systematic and avoidable; (2) facilitated and exacerbated by circumstances in which people live, work, and contend will illness; and (3) may be intensified by political, economic, and/or social influences [4]. Even in countries such as the U.S. that have economic power and several individuals with adequate resources, persons belonging to lower socioeconomic levels experience the worst health outcomes [4].

It is imperative that public health professionals, researchers, clinicians and policy makers embrace lead roles to bridge the gap between the rich and the poor concerning health issues, by promoting health equity and setting guidelines for global health initiatives. In order to address the plight of health inequities, social justice must be expanded to reach people on a larger scale which is more inclusive and less exclusive. We need leaders that will actively promote the CSDH three principles of action: (1) enhance daily living conditions in which people are born, grow, live, work, and age; (2) address inequitable distribution of power, money, and resources; and (3) accurately measure the issues, assess action plans, increase the knowledge base, create a workforce of persons trained in social determinants of health, and increase awareness about social determinants of health [5]. Moreover, one of the overarching goals for Healthy People 2020 is to “achieve health equity, eliminate disparities, and improve the health of all groups”. This can be accomplished with ethical and focused public health leaders at the helm. Using the public health approach which starts and ends with surveillance, indicates that it is appropriate to: (1) accurately define the health problem or opportunity; (2) determine the cause or risk factors involved; (3) determine what works to prevent or ameliorate the problem; and (4) determine how to replicate the strategy more broadly and evaluate the impact [5].

Addressing the multi-faceted health needs of ethnically and culturally diverse individuals in the United States is a complex issue that requires inventive strategies to reduce risk factors and buttress protective factors to promote greater well-being among individuals, families, and communities. There is growing diversity of various ethnicities and nationalities. There are significant changes in the constellation of multiple risk factors that can influence health outcomes, and it is imperative that we delineate strategic efforts that encourage better access to primary care, focused community-based programs, multi-disciplinary clinical and translational research methodologies, and health policy advocacy initiatives that may improve individuals’ longevity and quality of life. These issues have particular relevance for vulnerable and underserved populations, including African Americans, which have lower life expectancies compared to Caucasians in the U.S. [6].

## 2. Addressing Health Disparities from a Community Perspective

Community design assumes a major role in the overall health outcomes of community members. The built environment is defined as the “settings designed, created, modified, and maintained by human efforts, such as homes, schools, workplaces, neighborhoods, parks, roadways, and transit systems” [7]. Designs in the built environment, as well as natural landscapes, affect body structure and internal health as food environment and physical activity can be abundant or limited within one’s built environment. Design may affect accessibility to healthy drinking water or good quality air for breathing. Where one lives forms the basis for his/her health outcomes. It can enhance our quality of life, or it can adversely affect our very well-being. If a neighborhood lacks fundamental components within the built environment to support sufficient employment and education, access to healthy food options, sustainable active living space, and access to quality health care, then the risk of suffering from one or more chronic conditions exponentially increases for its residents [8].

Despite decades of research and programmatic enterprises, chronic medical conditions (such as diabetes and cardiovascular disease) remain a significant public health problem in the United States, especially for low income, racial and ethnic minority communities [9]. A myriad of social, structural, psychosocial, and environmental factors, including poor access to health care, food insecurity and lack of access to affordable healthy foods, lack of physical activity, and compromised mental and behavioral health, impact community members’ ability to participate in overall health-promoting behaviors, thereby exacerbating health outcomes [10]. Public health efforts to accelerate chronic disease prevention and reduce health inequities are increasingly focused on policy, systems, and environmental (PSE) approaches. Leading organizations such as the Centers for Disease Control and Prevention (CDC), Institutes of Medicine (IOM), the Robert Wood Johnson Foundation (RWJF), and the National Institutes of Health (NIH) have called for increased efforts at the state and local levels to advance such approaches. Changing policies and environments to promote active living and healthy eating require cooperation among diverse sectors [11]. Moreover, the CDC has highlighted the importance of coordination among multiple sectors as a key to successful efforts [12]. The IOM has emphasized the importance of engaging the non-health sectors in changing policies and environments to address chronic disease [13]. Collaboration should involve people or organizations from multiple sectors (e.g., planners, developers, media specialists, neighborhood residents, elected officials) and geographical strata (e.g., state, regional, local, neighborhood) [12]. Collaborative groups that promote stakeholder engagement and interaction have been associated with increased relevance, feasibility, and long-term sustainability of initiatives [14]. These groups have the potential to develop and maintain strategies to increase opportunities by leveraging resources, sharing knowledge, and building relationships [13]. The collaborative effort reflected in this proposal reflects a commitment to PSE approaches and the engagement of key stakeholders across sectors.

There are persistent gaps in many underserved, at-risk, and vulnerable communities for health promotion and disease prevention [15,16]. Social, emotional, and mental (SEM) problems can negatively impact an individual’s lifestyle behaviors that may increase their risk for a myriad of chronic disease [17]. One must consider the dynamic direct, indirect, and bi-directional relationships between SEM wellness and lifestyle behaviors such as physical activity [18], healthy eating [19], and tobacco-free living [20,21]. In particular, symptoms of a mental disorder, exposure to stressors, lack of social support, and the degree to which they believe behavior change is possible (self-efficacy) may harmfully impact: (1) receptivity to engaging in healthy lifestyle behaviors; (2) initiating behavior change; (3) resiliency when faced with setbacks and challenges; and (4) sustaining behavior changes on a long-term basis.

As health care reform is implemented, there is an opportunity to improve community health and health care. The crucial next step in advancing our scientific knowledge within selected populations is to establish multidimensional strategies that include communities, clinic systems, and community consumers’ collaboration that may bolster the potential for successes in the reduction of health disparities among vulnerable populations, including many African Americans. Specifically, part of the solution entails utilizing community based participatory approaches that: (1) leverage the experience and influence of community stakeholders to promote policy, environmental, and systems advocacy; (2) advance approaches for comprehensive integrated systems of care; and (3) improve community health leadership competencies and skills. Public health has an integral role in reducing health inequity, particularly concerning the distribution of resources through health education, creating a workforce of persons that target underserved communities, and increasing awareness about social determinants of health among bourgeoning professionals.

## 3. Community Engaged Approaches to Build Healthier Communities

### 3.1. Understanding Community Based Participatory Approaches

Historically, academic research in communities existed in which the academic institution received significant benefit; however, the community held no control of research projects and tended not to receive any benefit. Community-based participatory research (CBPR) is a research approach that emphasizes community-academic partnership and shared leadership in the planning, implementation, evaluation and dissemination of initiatives. Among the advantages of CBPR are strengthened neighborhood-campus relationships, improved research question relevance, enhanced research recruitment, implementation, collective dissemination, and mutual benefit for a diverse group of stakeholders [22–27].

The evolution and application of community based participatory research (CBPR) in communities has led to increased research participation and community ownership, globally. Conceptually, it is anticipated that through utilizing CBPR, outcomes will include not only answering a research question and reaping associated benefits, but also addressing community-identified social, economic or policy priorities [25]. One of the tenets of CBPR is the principle that researchers who want to conduct effective health research must invest time and resources in building partnerships with community-based organizations or neighborhood residents who are gatekeepers to establishing and maintaining community buy-in, ownership and sustainability. Ideally, community residents are equal or senior partners throughout the research process [26].

Previous meta-analyses and reviews have been conducted to understand CBPR, provide practical recommendations in its utilization, and to evaluate its research value, impact on health status and systems change [28]. Jagosh *et al.* [22] identifies contextual determinants of CBPR success that include the ability to collaboratively navigate conflict, negotiate and build consensus [29]. Among the results of successful partnerships are culturally and contextually tailored research, enhanced participant recruitment, and project sustainability. A recent meta-analysis of CBPR initiatives utilizing 46 instruments identified empowerment and community capacity measures among primary CBPR outcomes [30].

### 3.2. Benefits of Establishing a Community Coalition Board and Engagement to Build Healthier Communities

Establishing a governing body that ensures community-engaged research is challenging when: (1) academicians have not previously been guided by neighborhood experts in the evolution of a community’s ecology; (2) community members have not led discussions regarding their health priorities; or (3) academic and neighborhood experts have not historically worked together as a single body with established rules to guide roles and operations [31,32]. In the context of CBPR a community coalition board (CCB), composed of local stakeholders who serve and reside in prioritized communities adds substance to research and other health initiatives by providing local leadership and guidance on the most appropriate positioning of interventions, modes of community engagement for data collection, and access to neighborhood residents and leaders critical to effective public health initiatives [33,34]. Further, community residents’ lived experience as a group that may have experienced exploitation in research all the more requires that they not only hold a place at the research development and implementation table, but that their recommendations translate to action. Ideally, community residents should be equal or senior partners in relation to academic stakeholders on such boards, informing the development of the evaluation question, logic model, appropriate recruitment and retention strategies, and, most importantly, the translation of results to inform decision making, policy change, or subsequent research [33].

The Morehouse School of Medicine Prevention Research Center (PRC) was based on the applied definition of CBPR, in which research is conducted with, not on, communities in a partnering relationship faced with high levels of poverty, a lack of neighborhood resources, a plague of chronic diseases, and basic distrust in the research process as metropolitan Atlanta community members initially expressed their apprehension about participating in yet another partnership with an academic institution to conduct what they perceived as meaningless research in their neighborhoods. At the outset, the PRC created a governance model in which the community would serve as the “senior partner” in its relationship with the medical school and other academic and agency collaborators. The PRC is governed by a Community Coalition Board (CCB), to which all the identified partners belong, but community representatives hold the preponderance of power, literally putting them at the forefront of all CBPR and related approaches. Board members, including academic, agency, and neighborhood representatives, truly represent the community and its priorities. Academic representatives include the faculty and staff that are frequently engaged in carrying out the research service or training initiatives affiliated with the PRC. Agency staff (e.g., health department staff, school board representative) may not live in the community where they work, but their agencies serve the communities. Their input has value, but represents the goals and objectives of their organization, rather than the lived experience of a resident. Residents of the community—“neighborhood representatives”—are in the majority, and one always serves as Board Chair, as opposed to agency or academic members of the CCB. The PRC’s CCB serves as a policy-making board—not an “advisory board”, which has created an opportunity for community partners to have an active voice in directing the operations of and sustainability for the Center.

Central to establishing such a board was an iterative process of disagreement, dialogue, and compromise that ultimately resulted in the identification of what academicians needed from neighborhood board members and what they, in turn, would offer communities Not unlike other new social exchanges, each partner had to first learn, respect, and then value what the other considers a worthy benefit in return for participating on the board [35,36]. According to a former PRC CCB chair, community members allow researchers conditional access to their communities to engage in research with an established community benefit. Benefits to CCB members include the research findings as well as education, the building of skills and capacity, and an increased ability to access and navigate clinical and social services [36–41]. Benefits to board members in similar partnerships may also include dissemination of relevant and actionable research findings, the building of skills and capacity, and an increased ability to access and navigate clinical and social services. Among benefits to academic researchers are established community trust and relationships with partners beyond the community who have direct relation with the resources and partners that serve as local strengths and resources towards addressing health and social disparities and advancing health equity.

Critical to maintaining a community driven governance board are established bylaws that provide a blue-print for the governing body As much as possible, board members should be people who truly represent the community and its priorities. The differing values of academic and community CCB representatives are acknowledged and coexist within an established infrastructure that supports collective functioning to address community health promotion initiatives [33,42]. Lessons learned in CBPR community coalition board development and sustainability are detailed below:

- Engagement in effective community coalition boards is developed through multi-directional learning of each partner’s values and needs [38]
- Community coalition boards are built and sustained over time to ensure community ownership through established rules and governance structures
- Trust and relationship building are both central to having neighborhood and research experts work together to shape community-engaged research agendas
- Maintaining a community coalition board requires ongoing communication and feedback, beyond formal monthly or quarterly meetings, to keep members engaged

### 3.3. Strengthening Community-Academic Partnerships

To support building healthier communities, it is imperative to have community-academic partnerships which can garner a mutually beneficial experience. In the book, *Building Health Coalitions in the Black Community* [43], some of the building blocks of a strong partnerships include: clear identification of an issue/concern/topic, gaining support of key gatekeepers, stakeholders and agencies, establishing guiding principles including decision-making and action teams or committees, consensus building about the work to be accomplished, mapping of assets to enhance working relationships, effective communication and sharing of information, and performing continuous quality improvement/process evaluation of activities. Moreover, some of the characteristics of successful community-academic partnerships include:

- Attention to the fundamental tasks of long range planning, recruitment of members, and inter- and intra-coalition communication
- Monitoring of legislative and fiscal changes affecting the coalition and its members
- Leadership that emphasizes both task-oriented and interpersonal functions of the group
- Management of conflict within the coalition while maintaining its presence in the community
- Model whereby all members experience a sense of ownership and that they have impacted the action plan and implementation
- Diverse socialization opportunities (e.g., retreats, in-service training, workshops, *etc.*)
- Mentoring and training that focuses on developing leadership skills for members
- Aggressive fundraising and appropriate resource allocation

It is vital that both community members and academic institutions are mutually respected to avoid common reasons for coalitions and partnerships to fail, which include:

- Sabotage
- Interpersonal conflict and long standing feuds between partnering organizations
- Lack of genuine inclusion
- Hidden agendas of coalition members that can negatively influence other individuals
- Lack of group ownership
- Poor information/communication flow
- Lack of cultural competence
- Poor leadership

## 4. Significance of Ethical Leadership in Promoting Community Health

In the Institute of Medicine’s landmark report, *The Future of Public Health* [44] one major issue promoted was “the need for leaders is too great to leave their emergence to chance”. Moreover, we contend that principles espoused in the book, *Ethical Leadership: The Quest for Character, Civility and Community* [45] are essential to progressive innovative approaches and initiatives to build healthier communities. It is critical that leaders adopt leadership principles inclusive of: (1) insight—the importance of self-awareness, personal biases, and having empathy for others circumstances; (2) integrity—ethical governance and developing congruence between one’s own values and one’s actions; (3) synergy—learning the ability to work cooperatively and effectively with others in ways that empower individuals to use their gifts and make contributions that can benefit all parties; (4) sharing the “commitment to action”—developing the motivation to translate knowledge into action, foster buy-in and support, and to become actively involved in individual and collaborative efforts to foster personal and social change; and (5) impact—promoting positive civic engagement and social responsibility through an ethic of service and a concern for justice. In part, it will require focused training in these domains for community leaders to advance health equity. Examples of model leadership development programs are within the Satcher Health Leadership Institute (SHLI) at Morehouse School of Medicine (MSM). For example, SHLI’s Community Health Leadership Program, Health Policy Leadership Fellowship, Integrated Care Leadership Program, and Smart and Secure Parent Leadership Development Program have established pioneering strategies for preparing diverse community members, post-doctoral health professionals, physician leaders, and parents for tackling the myriad of complex and intricate health issues that plague underserved vulnerable communities.

Effective and ethical leadership is a critical key to success in the quest for building healthier communities. According to a first-ever study of U.S. medical schools in the area of social mission, MSM ranks #1 in the nation [46]. In order to encourage community health and ethical responsibility for future health care providers, researchers, and public health professional priority regarding leadership training is critical. There is leadership capacity in all of us; and we must help to develop that capacity because leadership matters. Leaders must be good learners, continually learning more about themselves, those they lead, and the cause or missions for which they work. Focused initiatives and cross-cultural collaborations will be achieved as we continue to transform the science of ethical decision-making and discovery in research, health promotion, and practice. U.S. based public health professionals, practitioners, research scientists, policymakers, community leaders, and individual consumers collectively have unique roles as thought leaders in the design, implementation, and evaluation of innovative strategies to promote community health and advance health equity.

## 5. Understanding Cultural Values and Implications of Planned Community-Based Activities

While socioeconomic, physical, and social environments can affect opportunities for healthy behaviors, the culture of communities must also be taken into account when developing interventions and seeking to engage communities for change. Research on health and health disparities demonstrate the importance of the built environment and the impact that systemic and structural changes can provide in relation to impacting health equality [47]; however the role of culture in engaging communities, designing interventions and implementation cannot be overlooked.

For example, an urban African American experience often lacks representation and input into community planning and infrastructure development as well as a lack of perceived power in engaging in decision-making about resource allocation. Discriminatory policies and practices tied to race/ethnicity and socioeconomic status have resulted in disinvestment in urban African American communities and resulted in underrepresented and disenfranchised residents [48]. Understanding the challenges and lack of engagement of urban communities in conjunction with the cultural mistrust is a critical but often overlooked aspect of research and intervention design. Research shows that when residents take an active role in improving neighborhood conditions, a positive effect on health results [49]. However, positioning health education as a permanent function requires the infrastructure for reliable and culturally congruent programming [50] that accounts for community input, non-traditional power centers, faith-based leaders and engagement of traditionally underrepresented segments of the community. Acknowledging the role of racism in health inequities and committing to addressing the root causes of health inequities is essential for establishing trust with community groups and in the development of successful culturally competent programming.

Despite the importance of addressing culture in community level interventions designed to improve health by addressing policies, systems, and the environment, there is a dearth of research focusing on culture and the built environment. Programs such as the Philadelphia Mural Arts Program [51] and Project ACHIEVE [50,52] are examples of community-engaged efforts that facilitate cultural tailoring of interventions to impact the physical environment and policy respectively. While there are many programs that operate within a community-engaged framework addressing population health, a gap remains in identifying best practices in attending to culture up front when designing place-based interventions [53].

Moreover, significant consideration that should be more supported in public health and a top priority of health delivery management teams is cultural competency training and education. According to the U.S. Census Bureau, non-Hispanic whites will comprise the numerical minority by 2050; and diversification is imperative for health care organizations to be more equipped to address cultural issues of varied patient populations that are served [54]. Cultural competence rests on a continuum and requires providers and public health professionals to reflect on their own identity, biases, and belief systems; and it is important to respect, understand, and accept other cultures [55].

In conclusion, to achieve the goal of lasting environmental change in the context of diverse communities, it is critical to: (1) engage neighborhood residents from the outset to build social capital; (2) use a comprehensive approach of community engagement which accounts for culture and historical inequities; and (3) make sustainability a priority.

## 6. Role of Policy, Systems, and Environmental Change Approaches to Building Healthier Communities

### 6.1. What Are Policy, Systems, and Environmental Change (PSE) Strategies?

Over the past decade, public health efforts to accelerate chronic disease prevention and reduce health inequities are increasingly focused on policy, systems, and environmental (PSE) approaches. PSE strategies employ modifications to written policies, established community/organizational systems, and built environments to improve access and opportunity for healthier behaviors [56]. PSE strategies also appreciate that interventions which target exo-system factors that influence individual health behaviors are more likely to lead to changes that are long-term and sustainable. Collectively, these approaches attend to the socio-ecological influences of health and human behavior that requires practitioners, researchers, policymakers and other stakeholders to understand psychological and social interactions at multiple levels of analysis and transactions between various networks and their relationships to outcomes. Community engagement is an important process and outcome involved in PSE approaches. It facilitates identification of community leaders’ knowledge and skills that should inform program and intervention components appropriate to the community context and designed to meet their health needs [57].

Policies, which refer to rules or procedures used to guide the execution of decisions and actions among individuals, exist at within organizations, agencies, and other governing bodies with the intention of producing positive outcomes [58]. Community institutions such as school districts, churches, non-profit organizations, health care organizations, commercial businesses and daycare centers develop and implement policies. Government bodies at the local, state, federal and international levels create policies that guide the activities of individuals and organizations within the jurisdictions they are responsible for governing. Additionally, policies are important for providing guidance to new partnerships and collaborations between entities such as community coalition boards and academic research teams that have come together to address a problem they can solve together more effectively than separate from each other.

Systems change involves changes made to the rules that various institutions, organizations, and agencies for example, that impact their operations and activities. These changes are made within existing infrastructures which may present challenges to successful implementation. For example, large systems that include thousands of individuals, have many smaller agencies or governing units within the larger system and are widely distributed geographically across a state, a country or around the globe, require changes to be carefully planned and executed to insure favorable outcomes [58]. Systems changes and policy changes are often complimentary and can support or hinder the health goals and objectives of the other depending multiple factors. Health care centers, schools, neighborhood clinics, and community service boards are examples of systems that can and often undergo changes that are designed to strengthen the health outcomes of individuals, families and communities they are responsible to serve.

Environmental change is imperative to strengthening communities. There are many types of physical environments that persons engage on a daily basis that can have a significant impact on their health outcomes including homes, community centers, prisons and grocery stores, for example. While a person may determine that they need to change their behavior to achieve a desired health outcome, examination of environments they frequent may reveal barriers or facilitators of that particular change that are not always readily apparent or observable. From sidewalks in communities designed to increase physical interactions between residents, to prisons that are designed to reduce the need for physical interactions to maintain control of incarcerated individuals, environmental changes can have lasting positive or negative effects on the health of persons within these spaces [58].

### 6.2. A Paradigm Shift

In The Institute of Medicine’s (IOM) landmark report—*The Future of Public Health*, one conclusion indicated was that the public health system and many of its policies involving assessment, service provision, program implementation and other functions was in disarray [44]. *The Future of the Public’s Health*, also published by the IOM in 2002 [59], expands this analysis and emphasizes the need for a population health approach, promotes interdisciplinary partnership and collaboration, and calls for a stronger public health infrastructure within government. There was explicit recognition that the policy, systems and environmental changes are critical in shaping the behaviors of individuals and health risks as well [59].

Throughout the late 1990s and 2000s, leading organizations such as the Centers for Disease Control and Prevention (CDC), Institutes of Medicine (IOM), the Robert Wood Johnson Foundation (RWJF), and the National Institutes of Health (NIH) have called for increased efforts at the state and local levels to advance such approaches. This is evidenced by key investments in community and population-level PSE initiatives made by several major entities including federal government agencies and private philanthropic organizations. Racial and Ethnic Approaches to Community Health (REACH) (1996–present), a national initiative administered by the Centers for Disease Control and Prevention to reduce racial and ethnic health disparities largely by promoting engagement between systems to impact health outcomes among disadvantaged populations. REACH program participants employ CBPR approaches to identify, develop and disseminate evidence based strategies to reduce and ultimately eliminate health disparities experienced by vulnerable communities of color. Strategies include a focus on proper nutrition, physical activity, and tobacco use and exposure include cardiovascular disease, diabetes, obesity and infant mortality. REACH awardees focus more directly on systems and environmental changes than policy change, but many achieve remarkable outcomes including lower smoking prevalence, increased intake of fruits and vegetables, and improving immunization rates [60]. Partnerships between governmental agencies such as school boards and health departments and non-governmental agencies such as churches, non-profit organizations, and businesses represent multi-sector collaborations that create program participants with knowledge, skills and the environmental conditions to make healthier lifestyle choices feasible.

The National Institutes of Health (NIH) has also supported key initiatives that utilize policy, systems, and environmental approaches to positively impact population health. The NIH’s Office of Behavioral and Social Science Research (OBSSR) brought together experts from a variety of disciplines including medicine, public health, nursing and social work to create a trans-disciplinary model of evidence based practice [61]. This body refined an evidence based model with an ecological framework that promotes change through engagement of interpersonal, organizational, community and public policy levels within practice and research settings. This effort is a great example of how system thinkers within a variety of disciplines collaborated to create a population-based approach to behavior change that was disseminated within and across disciplines, many of which have historically viewed individual-level change as normal and appropriate. Training modules have been developed for educators and evidence suggests that health care providers who have completed the modules demonstrate improvements in knowledge, attitudes and skills related to evidence-based practice [61].

### 6.3. Policy, Systems, and Environment Change Exemplars

While PSE strategies are diverse in their design and anticipated outcomes, several important exemplars have been recognized in the literature. Communities have achieved improved access to healthy food options through the development of healthy corner and grocery stores, community gardens, mobile food stores and pantries, and providing incentives for SNAP recipients to purchase fresh produce at locally based farmers markets [62–64]. PSEs that have been employed to increase opportunities for physical activity include Safe Routes to School initiatives, urban design and land use policies such as Complete Streets that promote active transportation, joint use agreements, and policies supporting the integration of brief bouts of physical activity into the standard routine of key community and organizational settings [65]. Reductions in the sale of tobacco products, tobacco use, and reduced exposure to tobacco byproducts (e.g., second hand smoke) have been achieved through the adoption of tobacco retail permitting, smoke-free business, school, and multi-unit housing policies [65,66]. Significant efforts have been made to systematically link high-risk community residents to preventive services and community-based wellness assets through: (1) employment of community health workers (CHWs) and other lay health promoters; and (2) leveraging of health information technology to identify high-risk patients and facilitate warm referrals [67–69].

### 6.4. Opportunities for Community Engaged Leadership in Policy, Systems, and Environment Changes

PSE strategies are nuanced and may require considerable investment in time and resources to achieve maximum impact. Effective, sustainable PSE strategies require collective action among diverse stakeholders, community buy-in, and constant communication to ensure all parties involved are operating from a unified action agenda. Thus, there are ample opportunities for community members and advocates to demonstrate leadership toward the successful adoption, implementation, and evaluation of PSE strategies. Lyn and colleagues [70] identify several key activities associated with PSE: (1) assess the social and political environment; (2) engage, educate, and collaborate with key stakeholders; (3) identify and frame the problem; (4) utilize available evidence; (5) identify policy solutions; and (6) build support and political will. Additional opportunities may arise through the PSE implementation process, and when evaluating PSE feasibility, impact on behaviors and attitudes, and effectiveness in mitigating deleterious health outcomes. We illustrate these crucial opportunities for community leadership by describing two emerging PSEs strategies being facilitated by the Morehouse School of Medicine REACH HI Initiative; Healthy Corner Stores and Complete Streets.

The REACH HI PSE initiative addresses existing PSEs that have contributed to the development of community environments that are barriers to healthy eating and physical activity. In the early 1960s federal transportation policies led to the construction and completion of the I-75/85 interstate highway connector, which cut through the heart of the City of Atlanta. The interstate divided downtown communities, destroying street grids and the connectivity of these neighborhoods. The impact of this imposing infrastructure and the community dissection it created has been disinvestment by businesses, including food establishments, and the loss of street connectivity that previously supported easier access to healthy foods, transit access, and physical activity. For example, from 1962 to 2006, Neighborhood Planning Unit (NPU)-V experienced an 86% decline in businesses; the number of businesses declined from 178 to 41. In 1962, NPU-V was home to 28 grocery/bakery/meat establishments and fifteen restaurants. By 2006, there were only four restaurants and five grocery/bakery/meat stores. As a result of the large loss of businesses and food establishments, corner stores emerged to serve as primary food sources for many in the community. These stores often offer food products that are energy dense but lacking in nutritional quality (e.g., high fat, high sugar). Efforts implemented in this initiative seek to counteract these challenges through conversion of corners stores to provide access to healthy foods and through policies that promote Complete Streets that are safe, connected, and supportive of physical activity.

Community-based participatory approaches were employed to conduct initial community health needs assessments and asset mapping project across several Atlanta NPUs in 2010–2011 and 2013. The assessments were led by a multi-sector coalition of Morehouse School of Medicine investigators, local community health organizations (e.g., United Way of Greater Atlanta), and a governance body comprised of local community residents and elected NPU chairs (Community Coalition Board). The most frequently cited health concerns identified through primary data included high blood pressure, diabetes and overweight/obesity. Among the common causes identified for these concerns were “stores without fresh fruits and vegetables”, “access and knowledge of healthy foods”, and “lack of affordable and healthy food and exercise options”. These concerns laid the foundation for the development of the Healthy Corner Stores and Complete Streets initiatives currently in effect. The Healthy Corner Store initiative seeks to recruit up to 21 local corner stores to enhance their provisions of fruits, vegetables, whole grain options, and low fat food options. The Complete Streets initiative intends to galvanize community support towards the advancement of Complete Streets policy adoption in five NPUs by 2017. All activities within both initiatives must be presented and endorsed by the local CCB prior to execution. Two community-based organizations are responsible for steering community engagement efforts and facilitating communications between community residents and academic investigators. Seasoned community health workers have been strategically employed to identify and map prospective corner stores; assess neighborhood infrastructure hazards (e.g., broken sidewalks, hazardous road conditions, *etc.*); identify existing Complete Streets and other infrastructure projects underway; and assist academic investigators with tailoring Corner Store community awareness and educational materials to best resonate with community stakeholders.

Although community leadership opportunities in employing PSE strategies are plentiful, some important key considerations must be acknowledged. PSEs must be in alignment with community stakeholders’ established needs, and community must be amenable to the proposed systems changes and environmental modifications being proposed. Cooperation across diverse sectors (with sometimes divergent agendas) is necessary to fully realize certain PSE strategies.

## 7. Toward Advancing Health Equity

Public health entities play a major role in reducing health inequities particularly by increasing resources for disadvantaged communities through various programs and by providing a trained workforce to educate these persons. For example, use of community health worker (CHW) and/or patient navigator models has increased in popularity around the globe since the 1980s, which has improved access to health care for underserved communities, supported efficiency in helping people with chronic illnesses to prioritize health management, engaged primary care services, and used preventive care services [71]. Section 5313 of the Patient Protection and Affordable Care Act (PPACA), Subtitle B—Innovations in the Health Care Work Force—recognizes CHWs as essential members of the health care delivery team; and Subtitle D—Enhancing Health Care Workforce Education and Training—indicated that the Centers for Disease Control and Prevention may be significant in facilitating community based efforts to promote health-seeking behaviors in underserved areas.

Health equity is “attainment of the highest level of health for all people” [9]. Lessons that continue to be learned from clinical practice, research, prevention initiatives, and advocacy to inform health policies each has unique yet complementary implications for approaches to improve health equity. There is value in examining successful models that have been implemented in various international regions that may inform models in the U.S. There is a need to more closely examine the significance and benefits of utilizing models of comprehensive, multi-disciplinary, culturally-tailored, patient-centered, and integrative health care delivery systems. For example, integration of behavioral health into primary care may yield positive outcomes and benefits at patient, provider, and clinic/system levels [72]. Also, this approach may help to improve access to quality health care in other countries, especially those with large rural populations that experience significant disparities in health and mental health. Furthermore, it may lead to gains in the development of conceptual frameworks to help reduce stigma in mental health help-seeking and treatment, as well as strategies for reducing disparities in health. Concerning research, innovative community-based, bio-medical, clinical and translational investigations are needed. These research studies must explore the complexities and intersection of multi-dimensional factors, bio-psycho-social issues, and cultural topics that help to elucidate emic and etic considerations about diverse groups. Better dissemination of research outcomes/findings to and from various local, national, and international communities by using inventive strategies will help to promulgate information to promote health. Furthermore, it is critical that prevention, intervention efforts, and health educational programs use bi-directional science discovery, evidence-based models, and intentional community engagement to encourage behaviors and practices that advance improvements in health. Working collaboratively with scholars, researchers and public health care professionals from international communities *versus* simply gathering data from their communities is a critical step in nurturing trust, strengthening credibility, and building global partnerships. Another vital ideal to consider for improving health equity is advocacy and strategic efforts to inform health policies. We have a responsibility to respond when: (1) an issue/topic (*i.e.*, health literacy) is identified but there is no policy to address it; (2) a policy is in place but it needs modification because it is ineffective or has yielded undesired outcomes; (3) a policy is in place but there are barriers to implementation (*i.e.*, health information technology in underserved communities); and (4) gaps that exists between science, policies, and cultural norms that deem the conducting impact analyses (*i.e.*, breastfeeding in the workplace).

Community engaged policy, systems and environmental approaches to improving the health of communities belong to an evolving public health approach that recognizes the importance of focusing on population health. As PSE approaches began to emerge in the late 1990s, particularly within public health, increased recognition and acknowledgement of forces that impact individual health behaviors and outcomes was embraced by stakeholders in medicine, public health, behavioral health and other sectors. This shift in thinking about how to create the conditions that support healthier communities through PSE approaches was supported by local, regional and national government agencies, faith-based, education, NGOs, and other organizations. Partnerships were formed and implementation science was developed to create an evidence base that revealed positive outcomes at the individual, family, and community level in a variety of areas including cardiovascular disease, obesity, diabetes, and hypertension.

We acknowledge that there are challenges to successful implementation of PSE approaches to pressing public health problems such as limited resources and funding. Limitations in available resources may present barriers at various levels for private and public sectors. Moreover, community needs may be identified, yet significant funding to support changes that could be sustainable are difficult to achieve. However, communities press forward, identifying creative and innovative solutions that maximize the skills, knowledge and experience emerging from partnerships that are community-based, egalitarian and promote consensus building. The ultimate goal of community-engaged approaches framed by PSE approaches under ethical leadership is improved community health. Increased utilization of focused, multi-dimensional, inter-sectoral strategies creates the opportunity for a larger positive impact on vulnerable and disadvantaged communities. Leadership that combines evidence based research and programming activities with a collaborative partnership with community members forms the basis of effective mechanisms to build healthier communities. Moreover, developing culturally centered tools and providing communities with educational resources to bolster knowledge and a sense of ownership of their communities, facilitates sustainability such that communities are empowered and mobilized.

Ethical leadership for community health promotion is an integral and central component of addressing health inequities; and stimulating positive change among policy makers and decision-makers. Perhaps, providing a cost-effectiveness and/or cost savings argument that can simultaneously strengthen communities on a systemic level that builds a sustainable infrastructure is one strategic method. This may be particularly relevant concerning the equitable distribution of resources to support health education, creating a workforce of persons that target underserved communities, increasing awareness about the role of social determinants of health among bourgeoning professionals, and working collaboratively with communities. It is imperative that we actively embrace the opportunities before us to respond to Dr. Martin Luther King’s proclamation to the Medical Committee for Human Rights in 1966 that “of all the forms of inequality, injustice in health care is the most shocking and inhumane” which starts with building healthier communities.

## 8. Conclusions

Researchers, public health professionals, clinicians, community members, and policy makers have distinct responsibilities to ensure the health and well-being of individuals, families, and communities. Collectively, through integrity-ethical based leadership, we can promote the reduction health disparities and advance health equity.

## Abbreviations

CBPA: Community-Based Participatory Approach
CBPR: Community-Based Participatory Research
CCB: Community Coalition Board
CDC: Centers for Disease Control and Prevention
IOM: Institute of Medicine
NIH: National Institutes of Health
NPU: Neighborhood Planning Unit
PRC: Prevention Research Center
PSE: Policy, System, and Environmental
RWJF: Robert Wood Johnson Foundation
